# Enhanced structure and function of human pluripotent stem cell‐derived beta‐cells cultured on extracellular matrix

**DOI:** 10.1002/sctm.20-0224

**Published:** 2020-11-04

**Authors:** Reena Singh, Louise Cottle, Thomas Loudovaris, Di Xiao, Pengyi Yang, Helen E. Thomas, Melkam A. Kebede, Peter Thorn

**Affiliations:** ^1^ Charles Perkins Centre, Discipline of Physiology, School of Medical Sciences University of Sydney Camperdown New South Wales Australia; ^2^ St Vincent's Institute Fitzroy Victoria Australia; ^3^ Computational Systems Biology Group Children's Medical Research Institute, University of Sydney Westmead New South Wales Australia; ^4^ Charles Perkins Centre, School of Mathematics and Statistics University of Sydney Sydney New South Wales Australia; ^5^ Department of Medicine, St Vincent's Hospital The University of Melbourne Fitzroy Victoria Australia

**Keywords:** basement membrane, diabetes, differentiation, glucose stimulated insulin secretion, human pluripotent stem cell‐derived beta cells

## Abstract

The differentiation of human stem cells into insulin secreting beta‐like cells holds great promise to treat diabetes. Current protocols drive stem cells through stages of directed differentiation and maturation and produce cells that secrete insulin in response to glucose. Further refinements are now needed to faithfully phenocopy the responses of normal beta cells. A critical factor in normal beta cell behavior is the islet microenvironment which plays a central role in beta cell survival, proliferation, gene expression and secretion. One important influence on native cell responses is the capillary basement membrane. In adult islets, each beta cell makes a point of contact with basement membrane protein secreted by vascular endothelial cells resulting in structural and functional polarization. Interaction with basement membrane proteins triggers local activation of focal adhesions, cell orientation, and targeting of insulin secretion. This study aims to identifying the role of basement membrane proteins on the structure and function of human embryonic stem cell and induced pluripotent stem cell‐derived beta cells. Here, we show that differentiated human stem cells‐derived spheroids do contain basement membrane proteins as a diffuse web‐like structure. However, the beta‐like cells within the spheroid do not polarize in response to this basement membrane. We demonstrate that 2D culture of the differentiated beta cells on to basement membrane proteins enforces cell polarity and favorably alters glucose dependent insulin secretion.

## Graphical abstract

Glucose stimulated insulin secretion is compromised in stem cell‐derived beta cells. In native islets, beta cell microenvironment play critical role in glucose sensing and stimulated insulin secretion. Reconstructing beta cell niche in vitro by introducing basement membrane proteins in planar culture, decreases basal insulin secretion, and enhances stimulation index in human pluripotent stem cell‐derived beta cells.

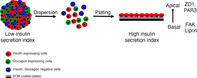

**Significance statement**

Current protocols are producing stem cell‐derived beta cells that secrete insulin in response to a glucose challenge. These cells are functionally close to normal beta cells, but refinements are needed in order to achieve full recapitulation of normal beta cell behavior. One promising avenue to enhance cell behavior is to look specifically at how beta cell structure and function is regulated by the environment of normal islets. In islets the basement membrane, secreted by vascular endothelial cells, has a direct effect on beta cell differentiation, survival, structure, and function. Each beta cell contacts the capillary basement membrane which then locally activates integrins and forms focal adhesions. The native beta cells then orientate with respect to the capillary to become polarized and to target insulin secretion to this capillary interface. This article reports on the effect of culturing human stem cells‐derived beta cells on basement membrane proteins, showing that this enforces structural polarity in the cells and significantly enhances glucose stimulated insulin secretion. The findings from this study suggest that adding environmental cues from the islet, such as basement membrane proteins, could be a promising route to enhance the function of human stem cells‐derived beta cells.

## 1 INTRODUCTION

Type 1 diabetes is caused by the autoimmune destruction of insulin‐secreting pancreatic beta cells.^1^ Treatment requires lifelong insulin injections and repeated monitoring of blood glucose throughout the day. Even with the best control, blood glucose commonly fluctuates outside the normal range^2^ and leads to long‐term complications that can have serious acute consequences, such as coma.^3^ Replacing the lost beta cells is in principle a cure as shown by the effectiveness of islet transplantation.^4^ However, issues with the quality and quantity of donor islets, as well as the consequences of long‐term immunosuppression, have driven the need for alternative approaches. Recent work on stem cells has made exciting progress and beta‐like cells, derived from human pluripotent stem cells (embryonic stem cell [ESC] and induced pluripotent stem cell [iPSC] refereed hereinafter as hPSC), can respond to glucose and secrete insulin.^5^, ^6^ Over the last 5 years, the differentiation protocols have been further refined^7^ and the cells produced are close to phenocopying the native beta cells.^8^, ^9^

However, current hPSC‐derived beta cells still contain and secrete less insulin than native beta cells, and they do not respond as well to normal blood glucose concentrations and have a limited lifespan.^8^, ^9^ The current focus in stem cell research has largely been at the level of the single cell and differentiation protocols target developmental switches to drive toward beta cell maturity.^5^, ^6^, ^9^ The results of these protocols are typically spheroids of a few hundred cells composed of beta‐like cells as well as other cell types. Despite improvements in differentiation methods, these cells have compromised metabolic glucose sensing and glucose stimulated insulin secretion (GSIS).^10^ Differentiated cells express comparable levels of metabolic enzymes^10^ and other gene signatures,^5^, ^7^, ^8^, ^9^ however, the microenvironment around each individual cell, within a spheroid, is not deliberately controlled and may or may not be optimal for cell function. This could be profoundly important because the normal islet environment around individual native beta cells has a dramatic effect both on their structure^11^, ^12^ and function.^13^, ^14^, ^15^, ^16^ Identifying the key aspects of these native environmental influences and recapitulating them in stem cell approaches could open new avenues in the goal to more faithfully phenocopy the function of native beta cells.

One aspect of the native islet microenvironment, the extracellular matrix, specifically the capillary basement membrane has a major effect on beta cell function. In native islets, the basement membrane is secreted by endothelial cells^11^, ^17^ and has a crucial impact on beta cell structure^15^ and function.^18^, ^19^, ^20^ In terms of structure, the beta cells contact the basement membrane only at the capillary interface.^11^, ^15^ This discrete contact area locally activates integrin‐dependent focal adhesions, drives beta cell polarization,^15^ and has multiple functional effects, such as proliferation,^11^, ^21^ insulin gene expression,^11^ and the control of insulin secretion.^19^, ^22^

The effect of basement membrane proteins on stem cell‐derived beta cells has not been investigated until very recently. The inclusion of basement membrane protein at specific stages of differentiation play role in beta cell fate determination^23^, ^24^ and function.^24^ However, these studies do not address the presence or the effect of basement membrane proteins on the function of fully differentiated cells.

Here, we employed modifications of the typical hPSC‐derived beta cells stem cell maturation protocols^5^ to produce spheroids of cells, including cells with a beta cell phenotype and other cell types, such as alpha‐like cells. Two different human pluripotent stem cell lines were used for this study; human iPSC (ATCC‐ACS‐1030) and human ESC *Ins* ^*GFP/W*^ (MEL1).^25^ We term both the ESC and iPSC pluripotent stem cell lines, hPSC. We demonstrate that differentiated hPSC‐derived spheroids do contain basement membrane, but it is present as unorganized structures within the spheroids with some beta‐like cells contacting the matrix and others not. We measure the subcellular organization of the cells within the spheroid and show that they have aberrant polarization and altered structural arrangement when compared to native beta cells. In further experiments, the differentiated cells were cultured as monolayers on basement membrane coated dishes. We show that the cells polarize with respect to the coated surface and the response to a glucose challenge is favorably altered when compared to cell spheroids with a decrease in basal insulin secretion and an enhanced secretory index.

We conclude that the addition of basement membrane to fully differentiated hPSC‐derived beta cells does drive changes in cellular structure facilitating the recruitment of insulin secreting machinery which enhances insulin secretion. This provides a platform for future work that aims to provide a better microenvironment for hPSC‐derived beta cells to enhance cell function and viability.

## 2 MATERIALS AND METHODS

### 2.1 Cell lines and culture conditions

Human iPSC obtained from American Type Culture Collection (ATCC‐ACS‐1030) and human ESC line *Ins* ^*GFP/W*^ (MEL1) was obtained from Murdoch Children's Research Institute, Victoria, Australia.^25^ Both the cell lines were maintained in mTeSR1 media (STEMCELL Technology, 85850) on Matrigel coated plates (Corning, 354277). Cells were passaged when reached 70% confluency. Cells were dissociated using TripLE Express 1X (Thermo Fisher Scientific; 12604021) and resuspended in mTeSR1 media containing 10 μM Rock inhibitor Y27632 (STEMCELL Technology; 72304). Cells were supplemented with fresh mTeSR1 complete media every day.

### 2.2 Differentiation of hPSC to insulin secreting beta cells

Cells were dissociated into single cell suspension and seeded at a density of 6 × 10^5^ cells/mL on ultra‐low attachment plates (Corning, COR3471) in mTeSR1 complete media supplemented with 10 μM Rock inhibitor (Y27632). Single cells clumped together to form embryoid bodies ranging from 65 to 241 μm in diameter. Differentiation was initiated after 48 hours for ESC and 72 hours for iPSC postdispersion as described in earlier protocols^5^, ^7^ (see Supplementary Information for details).

### 2.3 Glucose‐stimulated insulin secretion

Extracellular buffer (ECB) was prepared as follows: 140 mM NaCl, 5 mM KCl, 2.5 mM CaCl, 1 mM MgCl_2_, 5 mM NaHCO_3,_ 10 mM HEPES, pH 7.2. TNET lysis buffer composition is: 50 mM Tris‐HCl, pH 7.5, 150 mM NaCl, 1 mM EDTA, 1% Tx‐100, 10 mM Sodium pyrophosphate and freshly added protease inhibitor (Roche, 0463159001). Differentiated spheroids or differentiated cells as monolayer were washed with phosphate buffer saline (PBS) and starved for 1 hours in 3 mM D‐(+) Glucose ECB. Buffer was discarded and cells were further incubated in 3 mM D‐(+) Glucose ECB for another 1 hour. Buffer was collected as “basal secretion.” Cells were subsequently treated with 25 mM D‐(+) Glucose ECB for 1 hour and buffer was collected as “stimulated secretion.” Cells were then lysed in TNET buffer and saved as “total insulin content.” Homogenous time resolved fluorescence assay on the collected samples were performed using Cisbio Insulin ultra‐sensitive kit (62IN2PEG). Collected samples were diluted accordingly. Ten microliters of samples and standards were loaded in 384 well plates (Corning, 3824BC). Antibodies were added in a 1:1 ratio to the samples. Plates were sealed and incubated in dark overnight. Plates were run next day using Infinite F200 PRO (Tecan) plate reader and data were analyzed. DNA content in the lysed cells was measured using Quant‐iT PicoGreen Kit (Thermo Fisher Scientific; P7589) using FLUOstar Omega (BMG LabTech) plate reader.

### 2.4 Immunostaining of human pancreatic slices and hPSC‐derived beta cells

Native pancreatic tissue was sectioned into 150 μm slices using a vibratome. Pancreatic tissues were fixed with 4% paraformaldehyde (PFA) for 2.5 hours at 4°C. Differentiated spheroids were washed in PBS and fixed in 4% PFA for 1 hour at room temperature and used for whole mount immunostaining or for cryo‐embedding in optimal cutting temperature compound (Tissue‐Tek, 4583). Samples were cut in 10 μm sections. Cells on the monolayer were fixed in 4% PFA for 5 minutes on ice. All the samples were washed with PBS after PFA fixation and permeabilized in 0.1% Triton X‐100 (Scharlau; CAS No: 9002‐93‐1). Samples were blocked with PBS containing 5% donkey serum and 0.1% Triton X‐100 followed by overnight incubation in primary antibodies to a concentration of 1:100. Following primary antibodies were used for the staining purposes: Sox2 (Abcam; ab97959), Sox17 (Abcam; ab84990), Nkx6.1 (R&D Systems; AF5857), Pdx1 (R&D Systems; AF2419), Insulin (DAKO, A0564), Glucagon (Sigma, SAB4501137), Laminin (Abcam, ab11575), Collagen IV (Abcam, ab6586), Fibronectin (Abcam, ab2413), ZO‐1 (Thermo Fisher Scientific, 33‐9100), Liprin α1 (Proteintech, 14175‐1‐AP), E‐cadherin (Abcam; ab11512), Integrin β1 (BD Biosciences; 555002), PAR3 (Merck; 07‐330), and focal adhesion kinase (FAK; Cell Signaling Technology; 3285S). Next day, samples were washed in PBST (0.1% Triton X‐100) followed by incubation in secondary antibodies to a dilution of 1:200. Alexa Fluor secondary antibodies (Invitrogen) conjugated with 488, 546, 594, and 647 were used in different combinations for the visualization and imaging. Samples were finally washed and mounted in prolong antifade diamond (Thermo Fisher Scientific; P36961). All the images were acquired on Leica TCS SP8 STED microscope.

### 2.5 Flow cytometry of hPSC‐derived beta cells

Differentiated spheroids were dispersed using TripLE Express 1X (Thermo Fisher Scientific; 12604021) by incubating at 37°C and pipetting gently up and down until cells were dissociated into single cells. TripLE Express was quenched by adding S6 complete media. Cells were washed in PBS and fixed in 4% PFA on ice for 30 minutes. After fixation cells were washed with PBS followed by 1 hour's incubation in blocking solution on ice. Cells were further washed and incubated in blocking buffer containing primary antibodies for 2 hours on ice. Cells were washed three times using PBS followed by 1 hour's incubation in secondary antibodies on ice. Secondary antibodies were washed, and cells were resuspended in 1% fetal calf serum (FCS) in PBS for cell sorting/analysis. Cells were sorted using 10‐laser BD influx (BD Biosciences) and analyzed using FlowJo software.

### 2.6 Gene profiling using nanostring technology

For gene profiling human ESC, pooled samples from differentiation batches of human ESC‐derived spheroids and human islets from two different preparations were sampled. Total RNA was extracted using Isolate II RNA Mini Kit (Bioline; BIO‐52072). Concentration of RNA was analyzed using nanodrop. The precise quantity and quality of RNA were determined using Bioanalyzer RNA 6000 Nano assay (Agilent; 5067‐1511). The RNA Integrity Number value for the RNA samples were between 9.9 and 10. For the gene profiling of 50 selected genes, nCounter GX Custom CodeSets (NanoString; 116000001) was used. For each sample, 50 ng of RNA was hybridized with Reporter and Capture ProbeSet in a volume of 15 μL. Hybridization was carried at 65°C for 18 hours followed by a ramp down to 4°C. The hybridization samples were then loaded on nCounter SPRINT Cartridge (nanoString; 100 078) and run on nCounter SPRINT profiler. Data were analyzed using nSolver. Recommended quality control was performed. Background was subtracted using spiked negative controls. Absolute count of RNA was further normalized using the *POLR2A* housekeeping gene and plotted as heat map using GraphPad prism.

### 2.7 Human pancreatic islets

Cadaveric human islets were obtained from St Vincent's Institute. Consent was acquired for the use of islets in research. The study was approved by the Human Research Committee and The University of Sydney, Project No. 2017/042. Islets were cultured in RPMI‐1640 media supplemented with 10% FBS and 1% Pen‐Strep overnight (37°C, 95/5% air/CO_2_) before snap‐freezing in liquid nitrogen for RNA extraction.

**Table:** 

Donor	Age	Sex	Source
Islet preparation: 24 October 2018	37	F	St Vincent's Institute
Islet preparation: 19 August 2018	64	F	St Vincent's Institute

## 3 RESULTS

### 3.1 Human hPSC‐derived spheroids express monohormonal cells and exhibit insulin secretion upon glucose challenge

**FIGURE 1:**
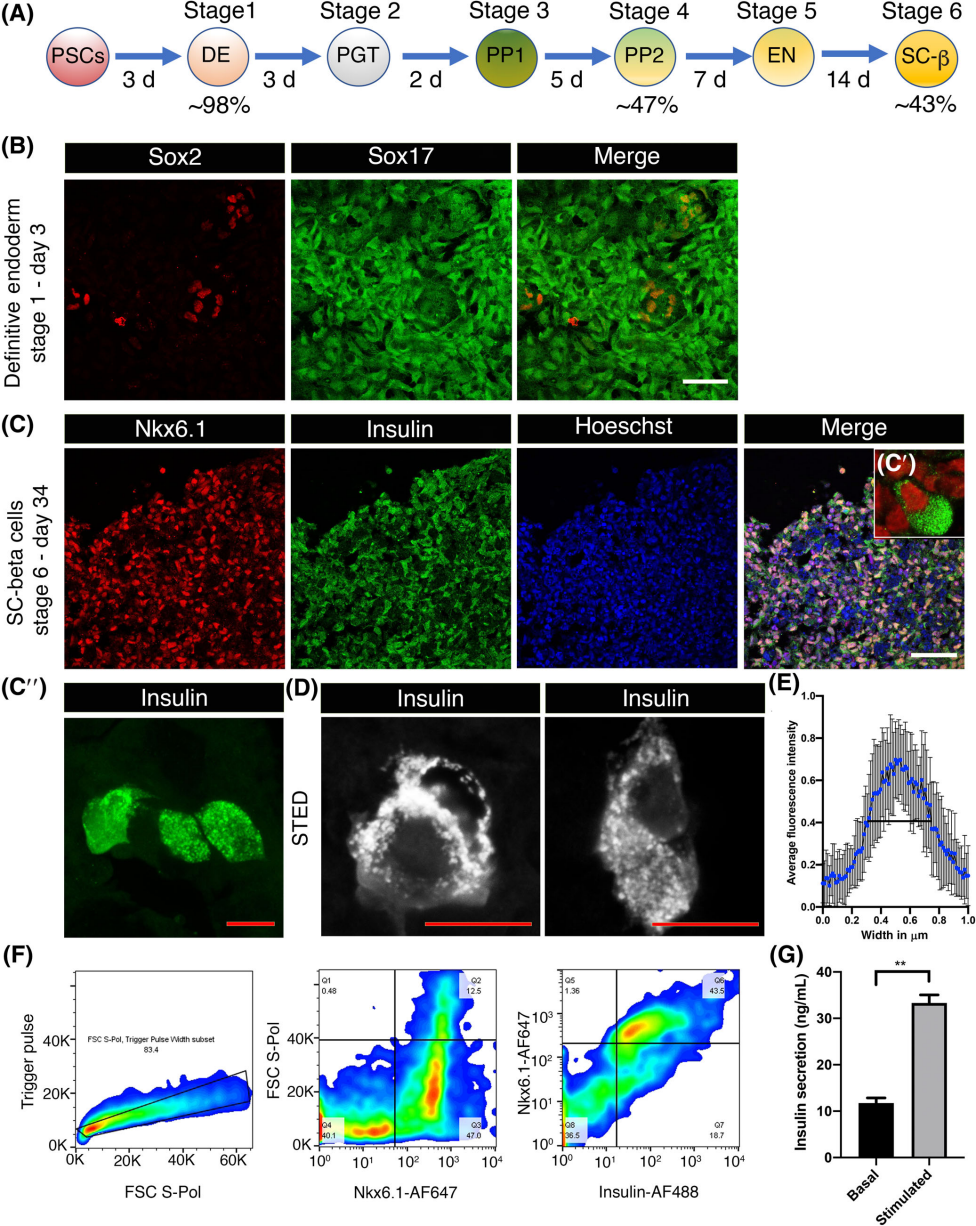
Human induced pluripotent stem cell (iPSC)‐derived beta‐cells secrete insulin in response to high glucose challenges. A, Schematic diagram showing differentiation of human iPSC/embryonic stem cell (ESC) to insulin expressing beta‐cells. Diagram depict six‐stages of differentiation: stage 1, definitive endoderm (DE); stage 2, primitive gut tube (PGT); stage 3, pancreatic progenitor stage 1 (PP1); stage 4, pancreatic progenitor stage 2 (PP2); stage 5, endocrine (EN); stage 6, stem cells derived beta cells (SC‐β). B‐D, Immunofluorescence data showing confocal (B,C″) and STED (D) images of differentiated human iPSC. B, By the end of day 3, the majority of the cells expressed DE marker Sox17 while very few cells retained stemness and expressed Sox2. C, At stage 6, day 34, majority of cells expressed insulin and co‐localized with Nkx6.1 expressing cells. While Nkx6.1 is expressed in the nuclear compartment, the expression of insulin is confined in the granular compartments (C′). C″, Higher magnification (×63) image of insulin expressing cells. D, High‐resolution STED images of insulin positive differentiated cells showing the expression of insulin in discrete granules localized in the cytoplasm. E, Analysis of n = 20 insulin containing granules. Insulin containing granules were on average of 0.4 μm diameter. F, FACS data of fully differentiated spheroids, stage 6, day 34. Approximately, 47% of cells expressed pancreatic progenitor marker Nkx6.1 and about 43% of that expressed insulin. G, HTRF assay showing glucose stimulated insulin secretion. Differentiated pooled iPSC‐derived spheroids were challenged with 25 mM (stimulated) glucose from a level of 3 mM (basal). White scale bars = 50 μm. Red scale bars = 10 μm. N = 3, Data are shown as mean ± SEM. Student's *t* test, **P = .002. d, days; FACS, fluorescence activated cell sorting; HTRF, homogenous time resolved fluorescence; STED, stimulated emission depletion microscopy

We established a stem cell differentiation protocol to produce spheroid cell clusters that contain beta‐like cells. Our protocol used human ESC and human iPSC and was based on published reports with a six‐stage, 34‐day‐long protocol.^5^, ^7^ Differentiation was carried out in suspension culture using cocktail of small molecules and recombinant proteins to maneuver cells through different stages; definitive endoderm (DE), primitive gut tube (PGT), pancreatic progenitor stage 1 (PP1), pancreatic progenitor stage 2 (PP2), endocrine (EN), and stem cell‐derived beta cells (SC‐β) (Figure 1A). The cell spheroids generated were 65‐241 μm in diameter (see Figure 5A). We dispersed the cells at each stage and used either flow cytometric analysis or immunocytochemistry to define the cell populations. The cells progressed from DE, with 98% of cells expressing Sox17 (Figure 1A,B), to 43% of the final resultant cells from hPSC expressing Nkx6.1 and insulin; both markers of beta cells (Figure 1C,C″,F). Super‐resolution imaging showed that insulin was expressed in punctate structures within the cell, with an average diameter of 0.4 μm, (Figure 1D,E) suggesting the formation of secretory granules.^26^

**FIGURE 2:**
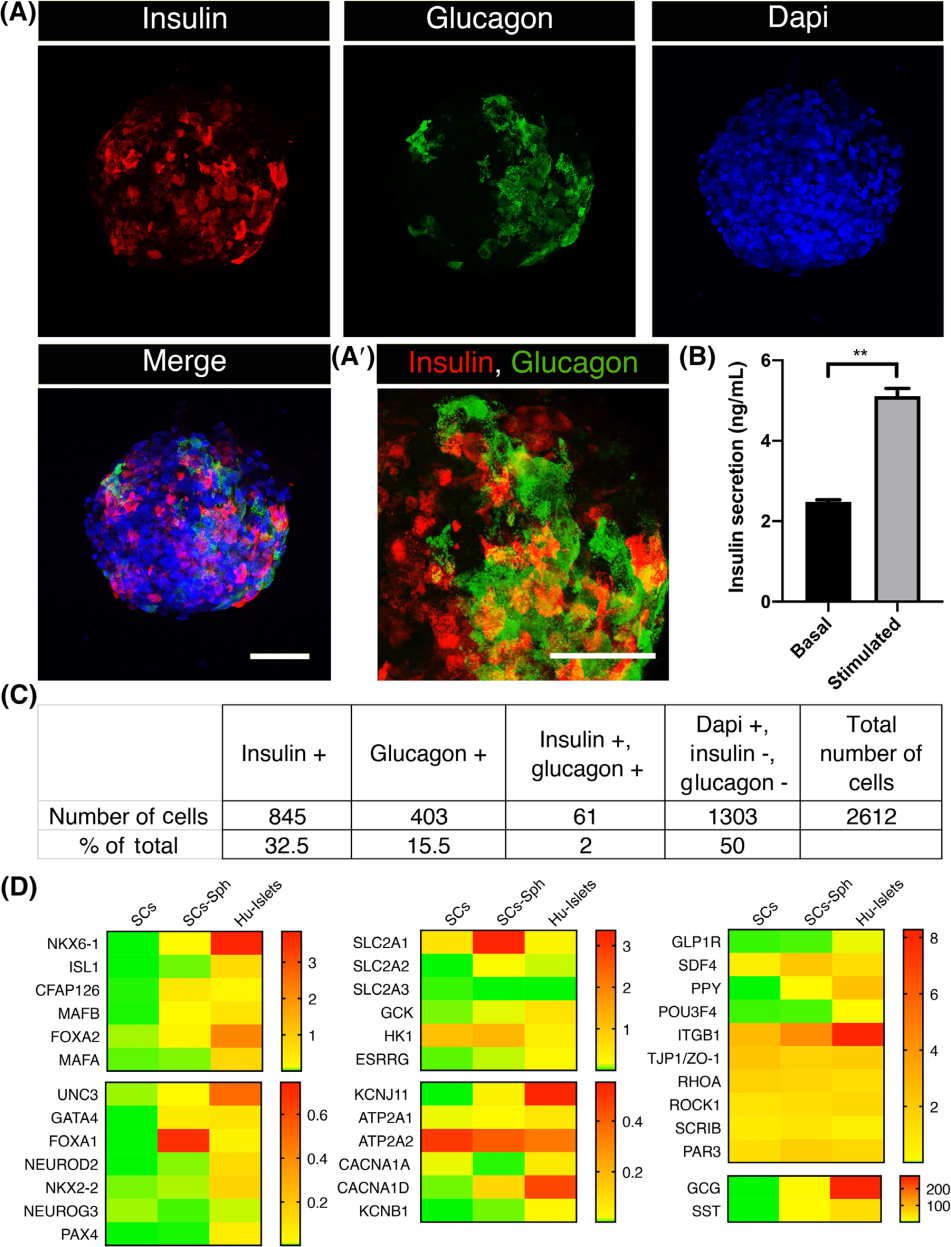
Differentiated human embryonic stem cell (ESC)‐derived spheroids predominantly harbor insulin‐producing monohormonal cells that express beta‐cell maturity markers, glucose transporters, metabolic enzymes, channel proteins, integrins, and polarity determinants. A, Z‐stack immunofluorescence confocal images of ESC‐differentiated spheroids. Insulin‐producing and glucagon‐producing monohormonal cells are predominantly present in the spheroids. A small proportion of cells are polyhormonal, expressing both insulin and glucagon. A′, Z‐stack higher magnification image showing the expression of insulin and glucagon. B, Insulin secretion assay showing a significant increase in the glucose stimulated insulin secretion in the differentiated ESC‐derived spheroids. C, Statistical analysis of immunostained differentiated ESC‐derived spheroids. Analysis of >2000 individual cells using ImageJ cell counter showing the percentage of insulin positive, glucagon positive, and insulin/glucagon double positive cells. D, Heat map showing transcriptional profiling data of human ESC (SC), differentiated ESC‐derived spheroids (SC‐Sph), and human adult islets (Hu‐Islets). Data generated using Nanostring technology on pooled samples of each category shows the expression of 37 different genes (raw counts of mRNA molecules) normalized to housekeeping gene *POLR2A*. The level of expression is graded from red (high) to green (low). The expression level of most of the genes aligns toward the human islets. Scale bars = 50 μm. N = 3, Data are shown as mean ± SEM. Student's *t* test, **P = .008

Since both glucagon secreting alpha and insulin secreting beta cells are derived from common endocrine progenitor and the presence of alpha cells in differentiated spheroids is reported,^8^ we characterized the human ESC‐derived spheroids using immunofluorescence to identify insulin and glucagon positive cells (Figure 2A). Composite Z‐stack images converted to RGB and different cell populations were marked manually and counted using cell counter Plugin on the ImageJ software. Our analysis shows 32.5% of cells expressed insulin, 15.5% cells expressed glucagon with only 2% expressing both insulin and glucagon (Figure 2A,A′,C). The presence of monohormonal cells is prerequisite for functional integrity. These spheroids were functional and demonstrated a 2.8 ± 0.28‐fold increase (mean ± SEM) for iPSC (Figure 1G) and 2 ± 0.11‐fold increase for ESC (Figure 2B) in insulin secretion in response to a switch from 3 to 25 mM glucose; values comparable to published reports for static insulin assays.^5^, ^9^

Further characterization was performed using transcriptional profiling of a panel of 50 genes to compare the characteristics of the hESC‐derived spheroids with those from human islets (Figure 2D). The panel included lineage markers, cell signature genes, metabolic markers, signal transduction, and polarity determinants. We found the hESC‐derived spheroids expressed beta‐cell lineage markers, such as NKX6‐1, CFAP126 (flattop), MAFB, FOXA2, UNC3 (urocortin 3), GATA4, FOXA1, and Nkx2‐2.^27^, ^28^ All were expressed above the levels of undifferentiated cells. However, there were differences in levels compared with human islets as has been observed in previous studies.^8^, ^29^ We observed high expression of glucose transporters, SLC2A1 (Glut1) and (SLC2A2) Glut2, metabolic enzymes such as glucokinase (GCK), potassium channels (KCNJ11, KCNB1), calcium channels (CACNA1A, CACNA1D), and calcium pumps (ATP2A1/SERCA1, ATP2A2/SERCA2b).

The gene signatures of the different endocrine cell types in islets were all expressed in hESC‐spheroids. These included: beta cell markers, Glucagon‐like peptide −1 receptor (GLP1R), SDF4; alpha cell markers Glucagon (GCG), POU3F4, delta cell marker somatostatin (SST), and gamma cell marker pancreatic polypeptide (PPY), suggesting that the differentiated spheroids harbor all the four endocrine cell populations.

We further characterized expression of signal transduction molecules such as integrin β1 (ITGB1), RHOA, and ROCK1 and presynaptic proteins such as PAR3, TJP1 (ZO‐1), scribble (SCRIB), and Liprin α1 (PPFIA1); which play critical role in integrin‐mediated cytoskeletal remodeling and glucose stimulated insulin exocytosis in beta‐cells^12^, ^14^, ^30^—all were found to be expressed in differentiated spheroids at comparable levels to human islets (Figure 2D).

We conclude that our differentiation protocol leads to a major population of monohormonal beta‐like cells that express many of the markers of beta cell identity and maturity. We show that the beta cells derived from both the stem cell lines (iPSC and ESC) are functional and secrete insulin in response to glucose. Finally, we demonstrate that the spheroids also contain other endocrine cells. However, while these spheroids are reminiscent of native islets, it is unknown whether they contain blood capillaries and the key biological effectors of the capillaries, the basement membrane protein.

### 3.2 Expression of basement membrane proteins form a mesh‐like structure in the differentiated spheroids

The differentiation protocol outlined above specifically aims to produce adult beta cells and therefore the structure of the spheroid forms spontaneously. Given native endocrine cells do not secrete basement membrane,^17^ we wanted to determine if any basement membrane proteins are present in the spheroids. Past work has shown some basement membrane proteins in the periphery of hESC‐derived pancreatic progenitor spheroids.^31^ Here, we wanted to determine if they are present in the mature differentiated hPSC‐derived spheroids and their spatial relationship with insulin‐expressing beta‐like cells.

**FIGURE 3:**
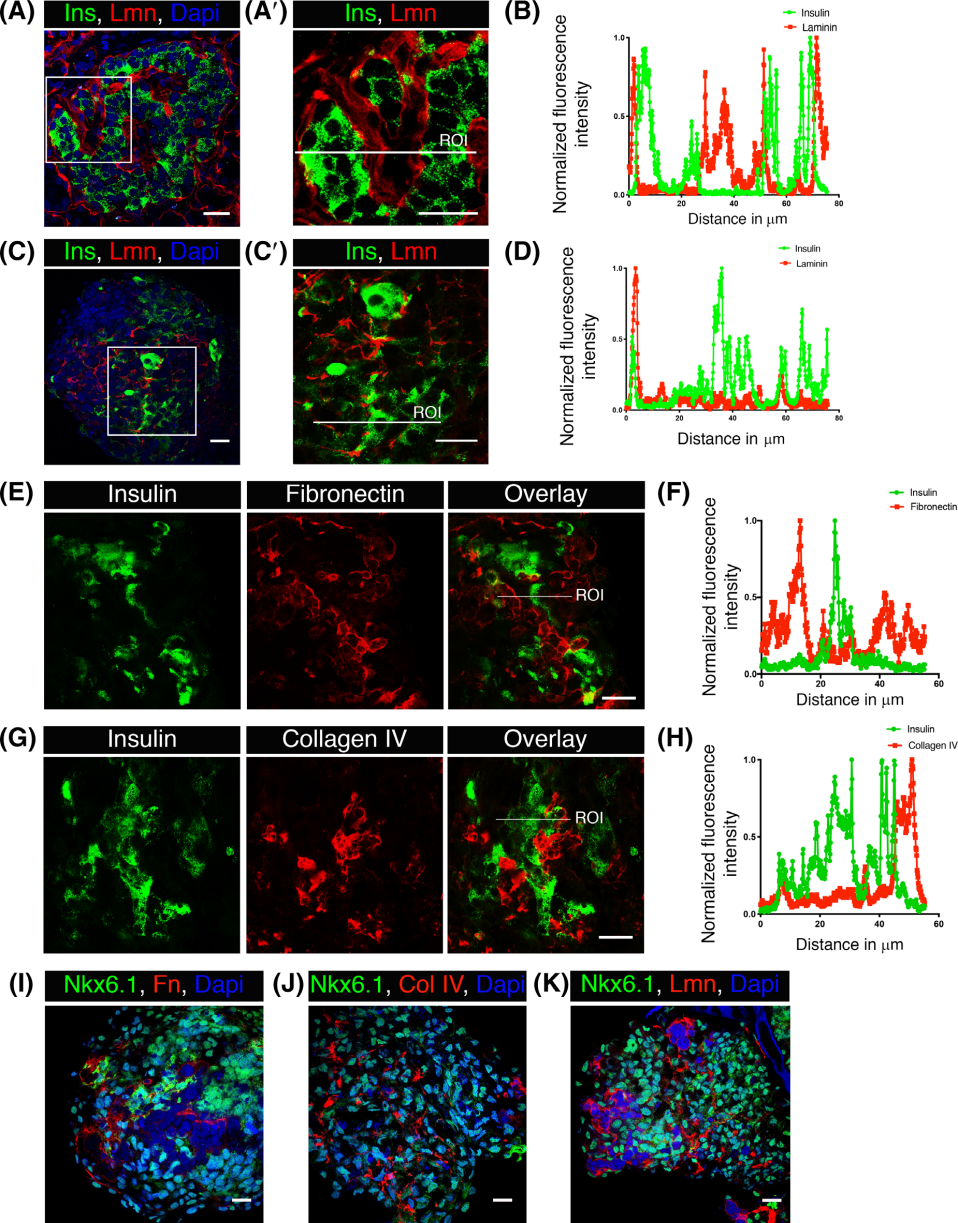
Differentiated spheroids express basement membrane proteins laminin, collagen IV and fibronectin forming a mesh‐like structure. A,A′, Immunofluorescence confocal images of human pancreatic slices. C,C′,E,G,I‐K, Immunofluorescence confocal images of embryonic stem cell (ESC) differentiated spheroids. AA′,C,C′,E,G, The expression of basement membrane proteins laminin, fibronectin, and collagen IV in red and insulin in green. B,D,F,H, XY line scan analysis at the region of interest (ROI) shown in the overlay images. Majority of beta cells make point of contact with basement membrane proteins as presented in line scan analysis of human islet (B). However, only fewer insulin expressing beta‐like cells interact with basement membrane proteins as presented in the line scan analysis of differentiated spheroids (D, F, H). I‐K, The expression of basement membrane proteins in red and pancreatic progenitor marker Nkx6.1 in green. Basement membrane expressing cells are distinct from pancreatic progenitor population. Scale bars = 25 μm

In mouse^12^ and human islets (Cottle et al submitted), the majority of the beta cells make a point of contact with basement membrane as is evident in Figure 3A,A′ and line scan analysis Figure 3B. To study the hPSC‐derived spheroids, we used immunofluorescence and 3D confocal microscopy to identify the presence and distribution of the basement membrane proteins, laminin, fibronectin, and collagen IV (Figure 3), all of which are present in human islets.^23^, ^32^ In differentiated cells, for all matrix proteins we observed a mesh‐like structure that ran throughout the spheroids (data from hESC are presented here). A proportion of beta cells do contact the basement membrane proteins as shown in the images (Figure 3C,C′,E,G) and measured using line scan analysis (Figure 3D,F,H). However, the majority of cells did not show any contacts with basement membrane proteins as is evident in Figure 3C‐H. It is difficult to determine which cells are secreting the basement membrane proteins but the regions surrounding the matrix proteins were largely negative for both Nkx6.1 (Figure 3I‐K) and insulin (Figure 3C‐H) suggesting that basement membrane proteins are secreted by cells other than beta‐like cells.

To further determine the origin of basement membrane proteins we analyzed single cell data of differentiated hESC (HUES8).^33^ Using bioinformatic tools (see Supplementary Information), six‐different cell types were identified; stem cell‐derived ‐alpha, ‐beta, ‐delta (SST^+^ HHEX^+^), enterochromaffin, endocrine cilia‐like (CHGA^+^ FOXJ1^+^), and nonendocrine cells. Normalized data were used for assessing the expression of a panel of marker genes in each of the six identified cell types (Supplementary Figure 1). These include basement membrane (COL4A2, COL4A5, FN1, LAMA1, LAMB1, and LAMC1), fibroblast (VIM and SEPRINH1), and endothelial (CD200) genes. Our analysis shows the predominant expression of basement membrane proteins in two different subsets of nonendocrine cells which also express fibroblast genes suggesting that the basement membrane identified in differentiated spheroids is secreted by stem cell‐derived fibroblasts (Supplementary Figure 1). Interestingly, expression of endothelial gene CD200 was not detected in basement membrane positive nonendocrine population. Other endothelial genes such as CD31 and CD34 were not quantifiable in the analysis (data not shown). The analysis also suggests the expression of one of the basement membrane protein LAMC1 in stem cell‐derived beta cells. However, the presence of only one chain perhaps is an indication of nonfunctional soluble protein.^34^ We conclude that basement membrane proteins are secreted by nonendocrine fibroblast‐like population of differentiated spheroids and contact some beta‐like cells. We next set out to determine if these contacts with the basement membrane influence beta‐like cell structural polarity.

### 3.3 Differentiated beta‐like cells do not show polar domain organization

**FIGURE 4:**
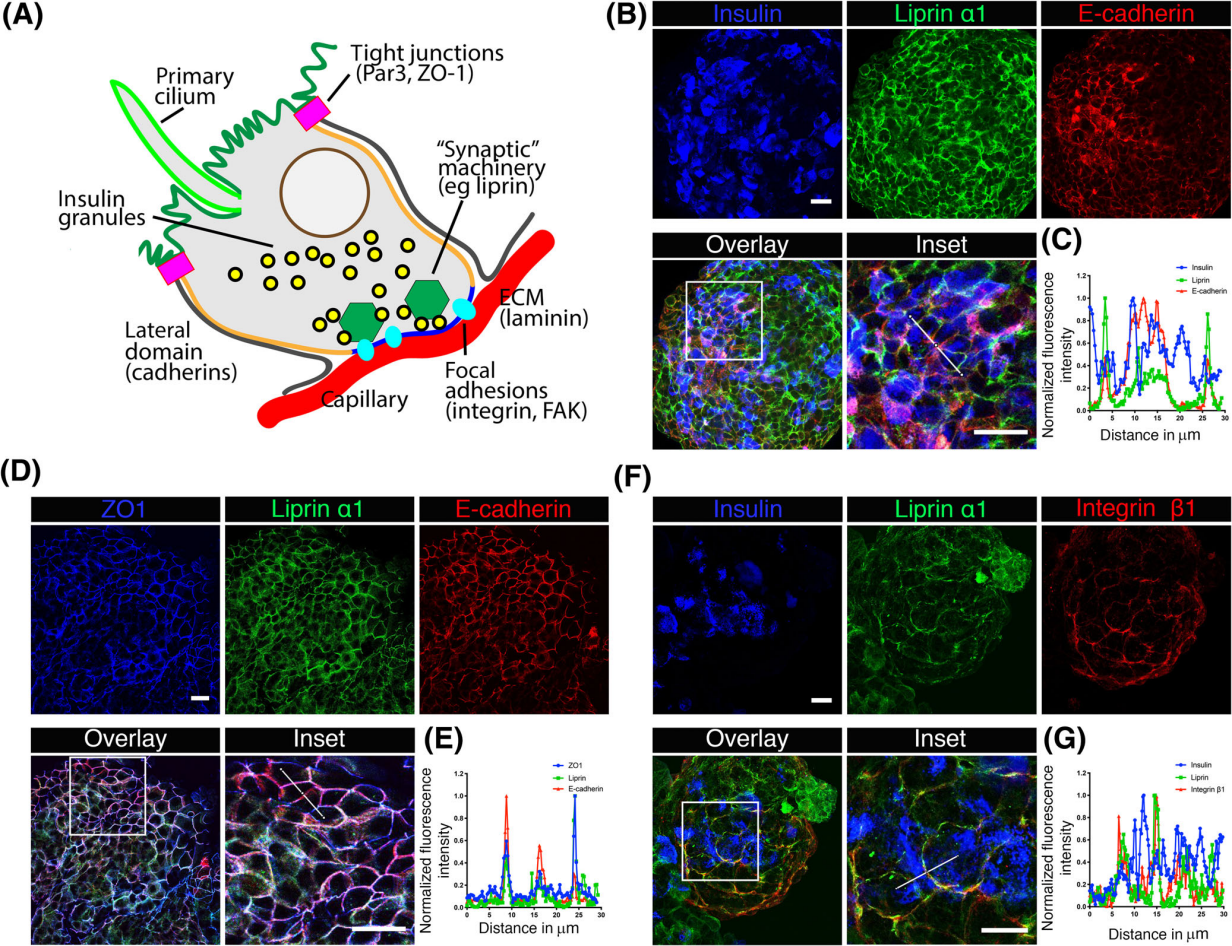
Human embryonic stem cell (ESC)‐derived beta‐cells express synaptic proteins but lack structural and functional organization in differentiated spheroids. A, Schematic diagram showing the spatial distribution of polar determinants and synaptic proteins in a native islet with respect to basement membrane (laminin) secreted by vascular endothelial cells. B,D,F, Confocal images of immune‐stained human ESC differentiated spheroids. A, Membrane expression of Liprin α1 (green) and E‐cadherin (red) in the insulin expressing (blue) differentiated beta‐cells. In the inset, distinct domains of Liprin α1 and E‐cadherin is seen in the insulin expressing cells. C, Line scan analysis on XY‐plane of insulin, Liprin α1 and E‐cadherin expressing cells. Liprin α1 and E‐cadherin expressions are either distinct or overlapped suggesting partial polarization. D, Shows the expression of ZO1 (blue), Liprin α1 (green), and E‐cadherin (red). E, Line scan analysis of inset showing expanded expression of ZO1 (apical domain) overlapping with Liprin α1 (basal domain) and E‐cadherin (lateral domain). F, Overlapping expression Liprin α1 and Integrin β1 is seen in insulin expressing cells. G, Line scan analysis of inset. Scale bars = 20 μm

The characteristic response of adult beta cells to the capillary basement membrane is integrin mediated cell orientation.^15^ This leads to the selective distribution of proteins associated with cell structure and function in the cell into three domains, namely apical, lateral, and basal.^12^, ^15^ Similar distribution of apical (PAR3 and ZO1), basal (integrin, FAK, and Liprin α1), and lateral domain proteins cadherin is seen in human pancreatic islets (Figure 4A) (Cottle et al submitted). To test if the hPSC‐derived beta cells in the differentiated spheroids also show domain segregation, we used immunofluorescence and 3D confocal microscopy to determine the subcellular organization of the tight junctional protein, ZO‐1 (apical domain), the pre‐synaptic protein Liprin α1 (basal domain), and E‐cadherin (lateral domain), specifically in the beta‐like cells (identified with insulin staining).

Immunostaining confirmed the presence of Liprin α1 (basal domain), integrin β1 (basal domain), E‐cadherin (lateral domain), and ZO‐1 (apical domain) proteins in the differentiated spheroids (data shown from ESC) (Figure 4B,D,F). Line scan analysis of Liprin α1 and E‐cadherin show that in certain regions of differentiated spheroids the two proteins are expressed in a mutually exclusive manner (Figure 4C) consistent with a location in different domains. However, in most parts of the differentiated spheroids there was lack of organization with overlapping expression of Liprin α1 and E‐cadherin (Figure 4B‐E; Supplementary Figure 2A,B), something not seen in native islets.^12^, ^14^ Similarly, the analysis of ZO‐1, Liprin α1, and E‐cadherin in spheroids showed colocalization of all these proteins at the cell membrane (Figure 4D,E; Supplementary Figure 2A,B), again something not seen in native islets. We did observe expression of integrin β1, overlapping with Liprin α1 (Figure 4F‐G). This is what is seen in native cells but, unlike native beta cells, this domain was not within a discrete subportion of the cell membrane but instead spread across much of the cell surface. We conclude that although the hPSC‐derived beta cells do express these proteins (also seen in the transcriptome data of Figure 2D), there is little evidence for polar organization and therefore it is unlikely that they have functional significance in the control of insulin secretion from the spheroids.

### 3.4 Interaction between beta‐like cells and basement membrane proteins favorably alters GSIS

Our data above show that the beta‐like cells in the differentiated spheroids are incorrectly polarized and have a disorganized structure (Figure 4B‐G; Supplementary Figure 2A,B) when compared to native beta cells in islets. We therefore wanted to constrain the cell environment around the hPSC‐derived beta cells to attempt to polarize the cells and recapitulate the organization observed in native beta cells in the intact islet. The simplest method is to culture cells on basement membrane coated surfaces which we have shown, for native cells, promotes local integrin activation at the contact surface and polarizes the cells.^15^

**FIGURE 5:**
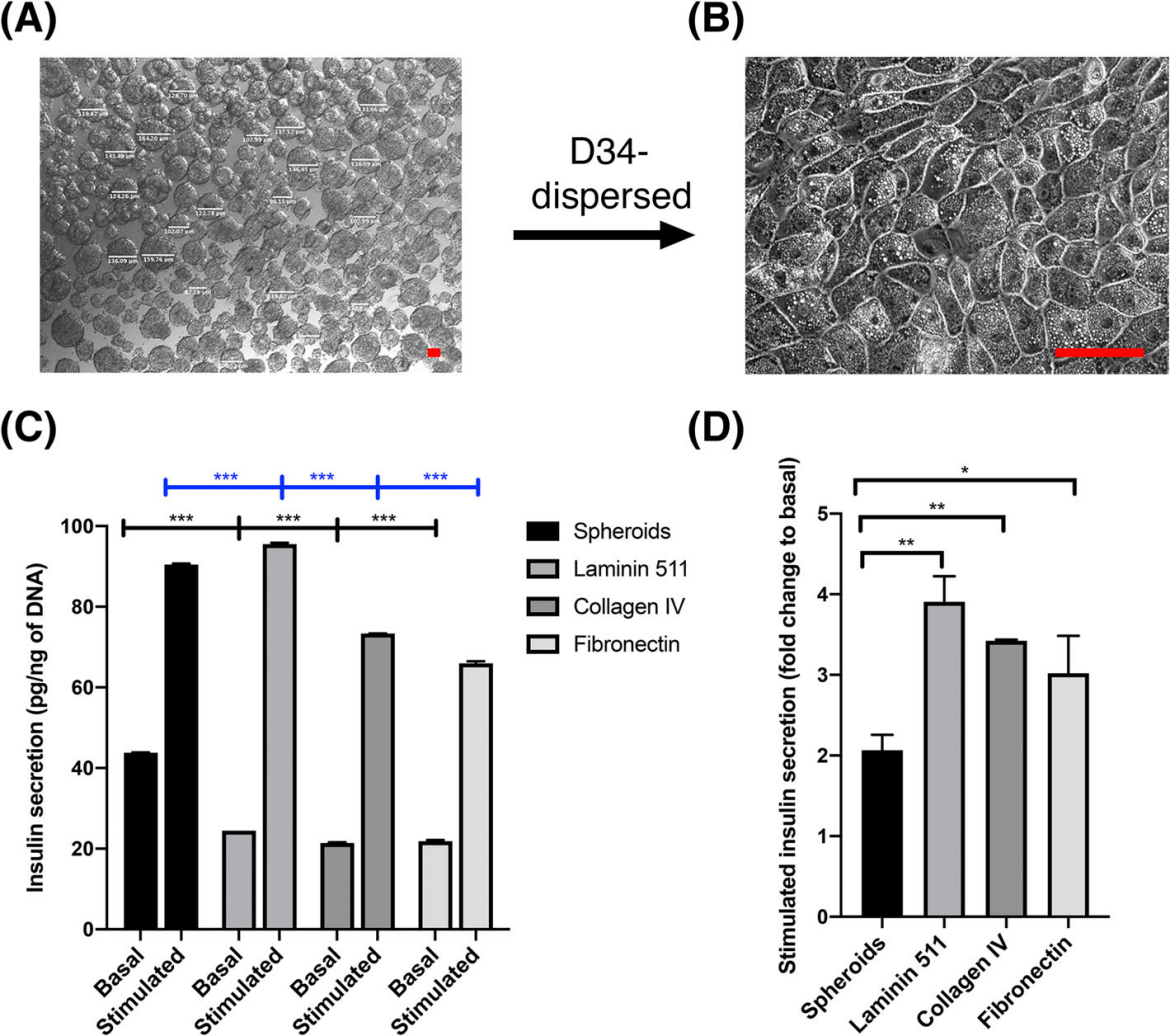
Human pluripotent stem cell (hPSC)‐derived beta‐cells show enhanced functionality on basement membrane proteins. A, Bright field image of human hPSC differentiated spheroids. The size of the differentiated spheroids ranges from 62 to 241 μm. B, Shows the monolayer image of hPSC‐derived beta cells plated on basement membrane protein. Tightly connected cells on the monolayer forming a cobblestone like structure are seen. C,D, Homogenous time resolved fluorescence (HTRF) assay showing glucose stimulated insulin secretion in differentiated spheroids and embryonic stem cell (ESC)‐derived beta‐like cells plated on basement membrane proteins laminin 511, collagen IV, and fibronectin. Downregulation of basal insulin secretion and upregulation of stimulated insulin secretion is seen on the basement membrane proteins. Basal = 3 mM, stimulated = 25 mM glucose concentration. N = 3. Data are shown as mean ± SEM. One‐way ANOVA, ***P < .001, **P = .002, *P = .033. Scale bar = 20 μm

We specifically chose to use cells from fully differentiated spheroids because basement membrane is known to control the fate of pancreatic progenitors.^23^, ^24^ Fully differentiated hESC‐derived spheroids were dispersed into single cells and plated on basement membrane protein coated dishes (see Supplementary Information). We observed cobblestone sheets of beta‐like cells in close contact with each other (Figure 5A,B). After 3 days of culture on basement membrane proteins (laminin 511, or collagen IV or fibronectin), GSIS assays were performed, exposing the cells sequentially to 3 mM (basal) and 25 mM (stimulated) glucose (Figure 5C,D). Compared to relatively high levels of basal insulin secretion in the differentiated spheroids (Figures 1G, 2B, and 5C), cells on basement membrane proteins showed a significant reduction in basal insulin secretion (Figure 5C). The stimulated insulin secretion (fold change from basal, stimulation index) was significantly upregulated on all the three basement membrane proteins (Figure 5C,D) with a maximum of 3.9‐fold increase with cells on laminin 511. We conclude that culture of cells onto basement membrane proteins significantly affects the insulin secretion and, by lowering the basal secretion, the fold increase is substantially improved when compared with spheroids.

### 3.5 Interaction between beta‐cells and basement membrane proteins polarizes the beta‐like cells

**FIGURE 6:**
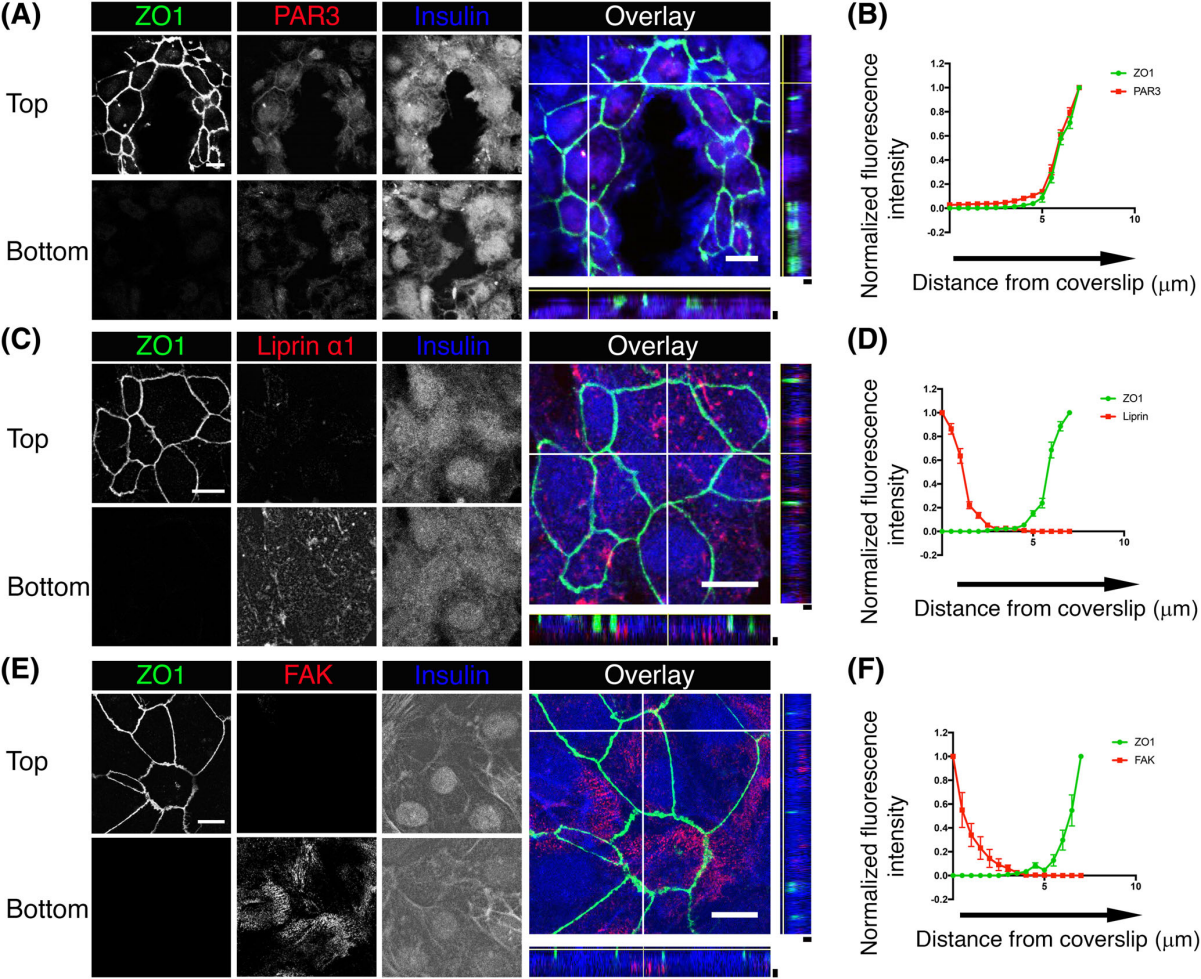
Polarization of human embryonic stem cell (ESC)‐derived beta‐like cells on basement membrane proteins. A,C,E, Z‐stack confocal images and the orthogonal sections of the differentiated cells plated as monolayer on laminin 511. The expression of ZO1 and PAR3 is seen enriched in the apical domain whereas the expression of Liprin α1 and focal adhesion kinase (FAK) is predominantly expressed closer to the coverslips in the basal domain of the monolayer cells. B,D,F, YZ‐line scan analysis of the orthogonal sections for N = 10 region of interests (ROIs). White scale bars = 10 μm. Black scale bars = 2 μm. Orthogonal sections data analysis are shown as mean ± SEM

We acknowledge that the benefits of cell culture into basement membrane might depend on multiple mechanisms, but here we wanted to test if cell polarization might be one of them. To analyze the structural and functional orientation of hESC‐derived beta‐cells cultured on to laminin 511 coated dishes we immunostained for some of the proteins characteristic of cell polarization. We observed ZO‐1 and Par3, both found at tight junctions, to be present in rings at the upper surface of the cell monolayers (Figure 6A,B), seen in the images and in line scans taken in the z dimension of orthogonal sections (Figure 6A‐D). This distribution is consistent with formation of an apical domain positioned away from the cell contact with the basement membrane protein. We next studied proteins characteristic of the basal domain, Liprin α1, and FAK. In both cases, we observed these proteins enriched where the cells interface with the laminin 511 coated coverslip (Figure 6C,E), opposite to the apical domain, labeled with ZO‐1, as shown in the line scans in the z dimension (Figure 6D,F). We conclude that the positioning of these proteins in hPSC derived beta cells is consistent with polarization of native beta cells in islets.

## 4 DISCUSSION

Remarkable progress has been made in generating insulin secreting beta‐like cells from stem cells in last decade.^5^, ^6^ For the purpose of scalability and robust differentiation, cells are maintained as spheroids in 3D culture, which means they will have endocrine to endocrine cell interactions that are expected to be analogous to those found in a native islet. However, the maturation and insulin secretion stimulation index of these cells are inferior compared to the human islets. Enrichment of beta cells and resizing of the clusters results into some improvement in the insulin secretion capacity,^8^ but they still have compromised glucose metabolism and insulin secretion.^10^

The identity of transporters and ion channels in beta cells that regulate glucose dependent control of insulin secretion is well known.^35^ Cell engineering approaches, using model cells, can recapitulate this framework and successfully lead to glucose‐dependent insulin secretion to an extent.^36^ However, normal beta cell function is strongly influenced by the islet environment^37^ and, importantly, this includes glucose‐dependent insulin secretion which shows an increase in basal secretion and decreased stimulated secretion in isolated cells compared to islets.^13^ Elevated basal insulin secretion and insufficient stimulated insulin secretion is a hallmark of immature human and rodent islets^38^, ^39^, ^40^, ^41^ as well as compromised islets.^42^, ^43^, ^44^ When the cells are cultured on basement membrane proteins, basal secretion goes down which is an indication of the effect of beta cell niche on structure and function. The microenvironment of beta cells in differentiated spheroids has not gained much attention and is not well understood. Recent studies also show that despite comparable levels of gene and metabolic enzymes expression, the microenvironment of the cells may play critical role in glucose sensing and insulin secretion.^10^

It is well established that one critical interaction of normal beta cells is with the basement membrane of the islet capillaries^11^, ^45^ with almost all beta cells making at least one point of contact.^12^, ^46^ This interaction with vascular basement membrane proteins fibronectin, collagen IV, and laminin 511 drives the spatial organization of beta cells within an intact islet with activation of integrin and formation of focal adhesions specifically at the capillary face.^18^, ^32^, ^47^, ^48^, ^49^ Focal adhesion kinase‐deficient beta cells exhibit impaired actin depolymerization and insulin secretion in in vivo rodent studies^48^ emphasizing the in situ significance of the basement membrane interaction for beta cells.

The importance of basement membrane proteins in endocrine differentiation has gained momentum in recent years. Integrin mediated mechanosignaling plays a critical role in fate determination of pancreatic progenitors.^23^, ^24^ Different basement membrane proteins may enhance or suppress endocrine differentiation depending upon mechanosignaling and actin cytoskeletal remodeling.^23^ Furthermore, fine tuning the cytoskeletal remodeling at specific stages of differentiation by the inclusion of basement membrane protein affects the quality of differentiated cells.^24^ None of these studies however, looked for the basement membrane proteins in differentiated spheroids and its effect on GSIS after maturation.

In this study, we show that basement membrane proteins are present as a diffuse web‐like structure in the differentiated spheroids. The origin of these basement membrane protein is not known but they unlikely to be secreted by the beta cells.^17^ The disorganized basement membrane web we observed in the spheroids (Figure 3C‐K) is therefore likely to come from nonendocrine cells that are a “by‐product” of the differentiation protocol. In human islets, basement membrane proteins are secreted by vascular endothelial cells. Vascular endothelial cells are derived from mesodermal lineage, therefore unlikely to be present in endodermal differentiation. However, we do acknowledge the presence of bi‐potent mesendoderm intermediate during ESC differentiation to DE^50^ and therefore the possibility of rudimentary endothelial‐like cells in the differentiated spheroids. Our bioinformatic analysis on single cell data (GSM3141957)^33^ (Supplementary Figure 1) and immunostaining on differentiated spheroids (data not shown) did not show the expression of endothelial genes CD31 and CD34 which needs to be further investigated. Our analysis also shows that basement membrane proteins in differentiated spheroids are coexpressed with fibroblast genes in nonendocrine cells (Supplementary Figure 1).

We further explored the structure of the hPSC‐derived beta cells within the spheroids and find that although they do express the polarity determinants and presynaptic proteins that are found in native beta cells, they are not organized, and the cells show no clear polarization. Structural polarization of native beta cells was identified some time ago^46^, ^51^ and the impact of beta cell polarity on structure and function is now increasingly recognized.^12^, ^15^, ^16^ This polarity is organized in a consistent manner with a basal region facing the capillaries, lateral regions where beta cells adjoin other beta cells and an apical region positioned at the opposite face.^12^ These distinct domains are further characterized by selective enrichment of functionally important proteins. The basal region which interacts with basement membrane protein to form integrin‐dependent focal adhesion^15^ is further identified by the localization of presynaptic scaffold proteins, such as ELKS, Liprin α1, RIM2, and piccolo^14^, ^52^ which regulate the stimulus secretion cascade^52^ to control insulin secretion^53^, ^54^ and target insulin granule fusion to the capillaries.^12^, ^14^ The lateral domain is enriched in cadherins^12^ which also regulate beta cell function.^55^, ^56^ The apical domain contains the primary cilia^12^ which again are thought to regulate cell function^57^ and is segregated from the rest of the cell by tight junctions identified by the presence of ZO‐1.^12^

Previous studies have shown benefit in the culture of isolated native cells basement membrane coated dishes in terms of increased insulin secretion that is dependent on integrin activation^49^ and leads to cell polarization with targeting of insulin secretion to the basement membrane.^15^

In our study, culture of hPSC‐derived beta cells on basement membrane protein also showed benefit. We observed polarization and a clear segregation between the apical and basal pole with enrichment of FAK and Liprin α1 toward the basement membrane and ZO1, PAR3 projecting further away marking apical pole. Significantly, we also show a functional benefit of culture on basement membrane protein. The stimulation index for hPSC‐derived beta‐cells varies anything between 2.2‐fold^5^ and 3‐fold stimulation index.^9^ By comparison, insulin stimulation index in static assays for human islets ranges from ~1.5 to ~10‐fold.^10^, ^58^ This range likely reflects differing qualities of the human islet preparations suggesting that for stem cell‐derived beta‐cells, we should be aiming for the upper end of the stimulation index. We show here that culture of hPSC‐derived beta cells on the basement membrane protein laminin 511 lifted the stimulation index from 2.8 in spheroids to 3.9‐fold. Stimulation index is only one measure of beta cell functionality, but our work indicates the addition of basement membrane proteins is a promising route to achieve better outcomes for regenerative medicine approaches. Destruction of vascular endothelial and extracellular matrix proteins during enzymatic islet isolation severely affects the quality of islet transplantation in human.^59^ Restoration of extracellular matrix and inclusion of basement membrane proteins enhance survival and function of transplanted cells.^60^, ^61^, ^62^, ^63^ Further approaches on modifying the niche of beta cells in differentiated spheroids such that the crosstalk between different endocrine cells and endocrine‐endothelial cells is established, should be investigated. This will help in recapitulating native islet environment and better functional outcomes for stem cell‐derived beta cells.

## 5 CONCLUSION

In summary, we show that hPSC‐derived spheroids contain disorganized basement membrane proteins which interact with a small proportion of beta‐like cells. We demonstrate the expression of polar determinates and synaptic proteins in differentiated spheroids which fail to polarize in the absence of well‐structured basement membrane. Our study concludes that addition of basement membrane protein to the fully differentiated hPSC‐derived beta cells in monolayer culture facilitate structural polarity and interestingly enhances GSIS. The increase in the fold secretion is attributed to lower basal secretion and increased stimulated secretion on basement membrane proteins as compared to spheroids. This study offers a novel strategy of modulating the microenvironment of stem cell‐derived beta cells for improving the functional outcomes of stem cell therapy for diabetes mellitus.

## CONFLICT OF INTEREST

The authors declared no potential conflicts of interest.

## AUTHOR CONTRIBUTIONS

R.S.: conceptualized, designed, and conducted experiments; L.C.: designed and conducted experiments; D.X., P.Y.: bioinformatic analysis on single cell data; M.K.: provided expertise and helped experimental design; T.L., H.E.T.: provided the human material and expertise; P.T.: designed the project and managed data analysis. All authors contributed to writing the manuscript.

## Supporting information


**Appendix** **S1:** Supporting information

## Data Availability

The data that supports the findings of this study are available in the supplementary material of this article.
