# Supplementary material for: Enhanced structure and function of human pluripotent stem cell‐derived beta‐cells cultured on extracellular matrix
Source: Stem Cells Transl Med. 2020 Nov 4;10(3):492–505. doi: 10.1002/sctm.20-0224 (PMC7900592; doi:10.1002/sctm.20-0224)
Supplement: Supplementary file 1 — Appendix S1: Supporting information [file SCT3-10-492-s001.pdf]

Supplemental Information

Enhanced structure and function of human Pluripotent stem cell derived beta-cells cultured on extracellular matrix

Reena Singh[1], Louise Cottle[1], Thomas Loudovaris[2], Di Xiao[4], Pengyi Yang[4,5], Helen E Thomas[2,3], Melkam A Kebede[1], Peter Thorn[1]

[1]Charles Perkins Centre, Discipline of Physiology, School of Medical Sciences, University of Sydney, Camperdown, NSW 2006, Australia

[2]St Vincent's Institute, Fitzroy, Victoria 3065, Australia

[3]The University of Melbourne, Department of Medicine, St Vincent's Hospital, Fitzroy, Victoria 3065, Australia

[4]Computational Systems Biology Group, Children's Medical Research Institute, University of Sydney, Westmead, New South Wales, Australia

[5]Charles Perkins Centre, School of Mathematics and Statistics, University of Sydney, Sydney, New South Wales, Australia

Correspondence to:
Dr Reena Singh,
Charles Perkins Centre, Discipline of Physiology, School of Medical Sciences, University of Sydney, Camperdown, NSW 2006, Australia

Email: Reena.singh@sydney.edu.au

The authors declare no competing interests.

Keywords
Human pluripotent stem cell-derived beta cells, differentiation, glucose stimulated insulin secretion, diabetes, basement membrane

SUPPLEMENTARY FIGURES

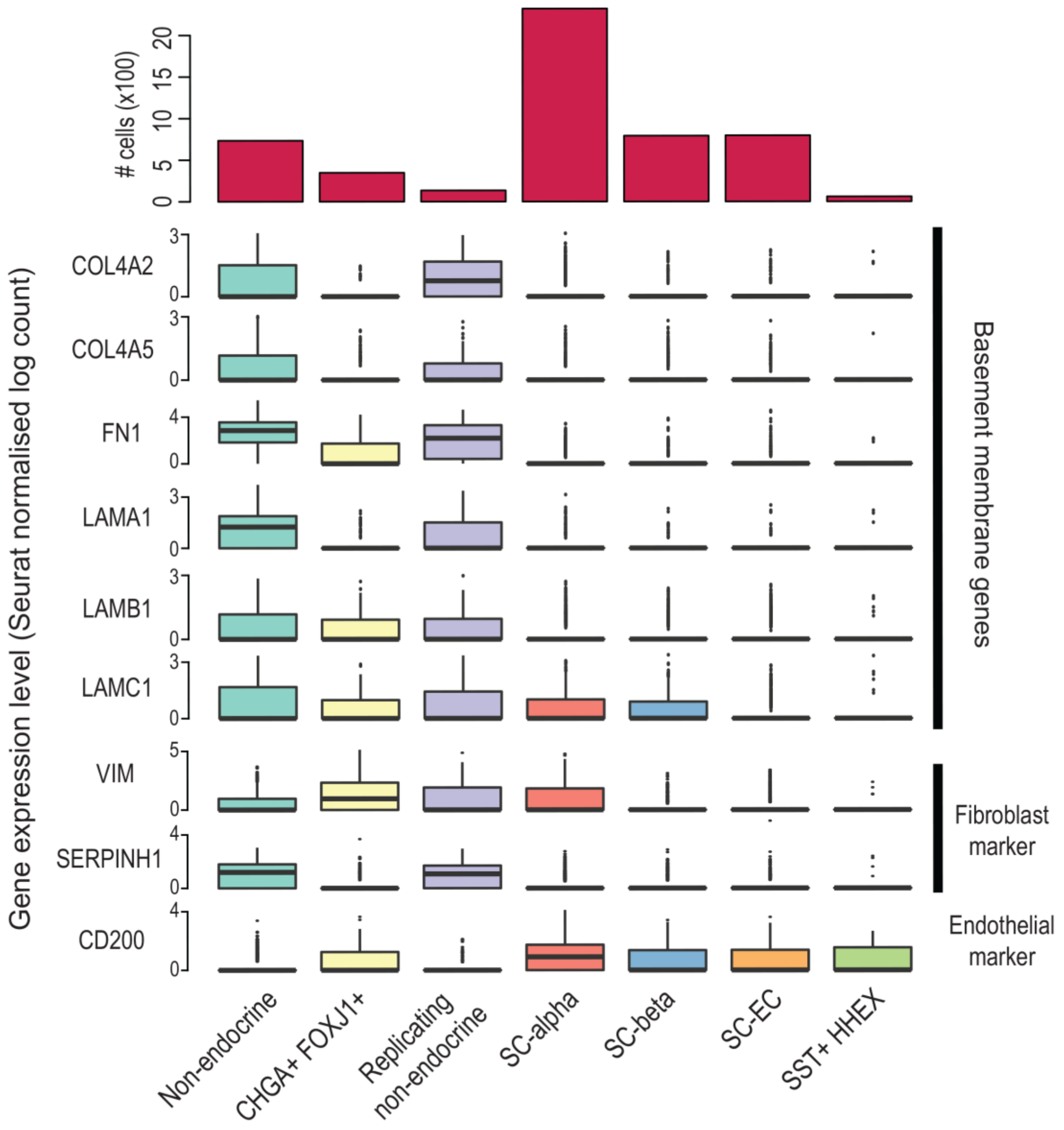

Figure S1. Basement membrane protein in differentiated spheroids is expressed by non-endocrine fibroblast-like cells.
Boxplot of single cell RNA seq profile for differentiated human ESCs (HUES8) (GSM3141957) [34]. The expression of marker genes for basement membrane, fibroblast and endothelial cells are shown in six-different identified cell types; stem cell-derived non-endocrine cells, CHGA+ FOXJ1+ endocrine cells, replicating non-endocrine cells, stem cell derived -alpha cells (SC-alphaCs), -beta cells (SC-betaCs), -enterochromaffin cells (SC-ECs), SST+HHEX+ delta cells (SC-deltaCs). Basement membrane genes include collagen type IV alpha 2 chain (COL4A2), collagen type IV alpha 5 chain (COL4A5), fibronectin 1 (FN1), laminin subunit alpha 1 (LAMA1), laminin subunit beta 1 (LAMB1) and laminin subunit gamma 1 (LAMC1). Fibroblast markers include vimentin (VIM) and serpin peptidase inhibitor clade H member 1 (SEPRINH1). Endothelial marker includes cluster of differentiation 200 (CD200). Gene expression level is plotted as Seurat normalised log count. Basement membrane genes predominantly co-expressed with fibroblast markers in the non-endocrine population.

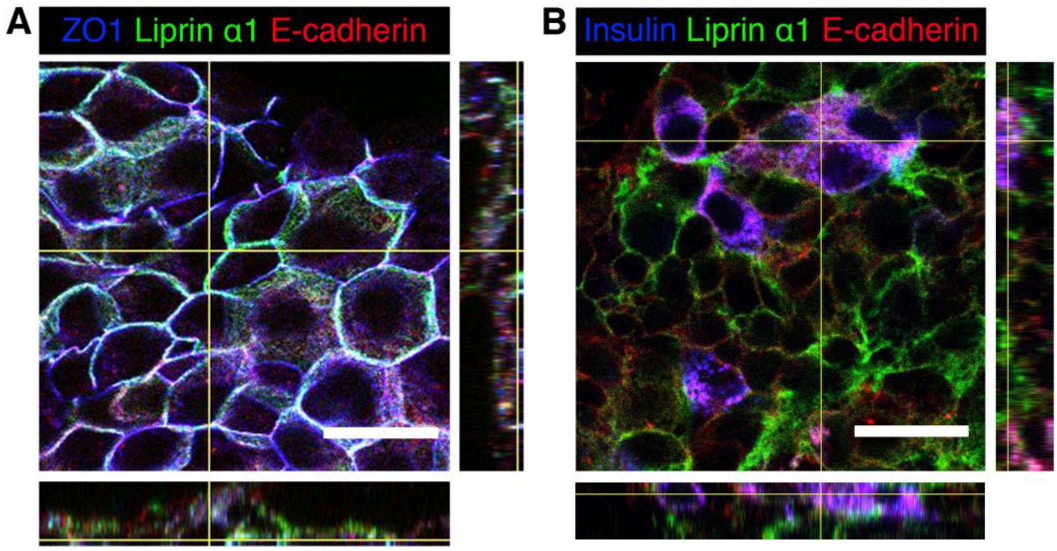

Figure S2. Human hPSCs-derived spheroids lack structural organisation.
(A, B) Orthogonal section of confocal images of ESC-derived spheroids. (A) Showing dis-organised expression of ZO1 (blue), Liprin α1 (green) and E-cadherin (red). (B) Showing partial polarisation for E-cadherin (red) and Liprin α1 (green) with insulin expressing cells (blue). Scale bars, 20 μm.

SUPPLEMENTARY MATERIALS AND METHODS

S1.1 | Differentiation of human hPSCs to insulin secreting beta cells

A six-stage, 34-days long differentiation was carried out using following formulations:
Stage 1 (3 days): S1 media supplemented with 100 ng/ml Activin A (StemCell Technology; 78001.1) and 3 µM Chir99021 (StemCell Technology; 72054) for 1 day. S1 media supplemented with 100 ng/ml Activin A for 2 days. Stage 2 (3 days): S2 media combined with 50 ng/ml of Keratinocyte Growth Factor (KGF) (StemCell Technology; 78046.1). Stage 3 (2 days): S3 supplemented with 50 ng/ml KGF, 0.25 µM SANT-1 (Sigma-Aldrich; S4572), 2 µM Retinoic Acid (RA) (Sigma-Aldrich; R2625), 200 nM LDN193189 (day7 only) (Sigma-Aldrich; SML0559) and 500 nM PdBU (Sigma-Aldrich; P1269). Stage 4 (5 days): S3 media containing 50 ng/ml KGF, 0.25 µM SANT-1, 100 nM RA, 10 µM Y27632 and 5 ng/ml Activin A. Stage 5 (7 days): S5 media supplemented with 0.25 µM SANT-1, 100nM RA, 1µM γ-secretase inhibitor XXI (StemCell Technology; 73954), 10 µM Alk5i II (StemCell Technology; 73794), 1 µM T3 (Sigma-Aldrich; T6397) and 20ng/ml Betacellulin (In Vitro Technologies; RDS261CE010CF) for 4 days following by 25nM RA, 1 µM γ-secretase inhibitor XXI, 10 µM Alk5i II, 1 µM T3 and 20 ng/ml Betacellulin for 3 days. Stage 6 (15 days): S6 media containing 10 µM Alk5i II and 1µM T3 with media change every alternate day.

The base media used at each step is outlined below:
S1: MCDB 131 (Life Technologies; 10372019) supplemented with 8 mM D-(+) Glucose (Sigma-Aldrich; G8270), 2.46 g/litre $NaHCO_3$ (Sigma-Aldrich; S5761), 2% Bovine Serum Albumin (BSA) (Sigma-Aldrich; A8806), 1:50,000 dilution Insulin-Transferrin-Selenium-Ethanolamine (ITS-X) (Life-Technologies; 51500056), 2 mM Glutamax (Life-Technologies; 35050061), 0.25 mM Vitamin C (Sigma-Aldrich; A4403), 1% penicillin-streptomycin (Pen-Strep). S2: MCDB 131 supplemented with 8 mM D-(+) Glucose, 1.23 g/litre $NaHCO_3$, 2% BSA, 1:50,000 dilution ITS-X, 2 mM Glutamax, 0.25 mM Vitamin C, 1% Pen-Strep (ThermoFisher Scientific; 15140122). S3: MCDB 131 supplemented with 8 mM D-(+) Glucose, 1.23 g/litre $NaHCO_3$, 2% BSA, 1:2,00 dilution ITS-X, 2 mM Glutamax, 0.25 mM Vitamin C, 1% Pen-Strep. S5: MCDB 131 supplemented with 20 mM D-(+) Glucose, 1.754 g/litre $NaHCO_3$, 2% BSA, 1:2,00 dilution ITS-X, 2 mM Glutamax, 0.25 mM Vitamin C, 1% Pen-Strep, 10 µg/ml Heparin (Sigma-Aldrich; H3149). S6: CMRL 1066 supplemented media (Focus Biosciences; 99-603-CV) containing 10% FBS, 1% Pen-Strep.

S1.2 | Culturing differentiated cells on basement membrane proteins

Tissue culture treated 24-well plates (Corning Costar; 3524) and two-well chamber slides (Lab-Tek; 177429) were coated with basement membrane proteins laminin 511 (BioLamina; LN511-0502), collagen IV (Sigma; C5533) and fibronectin (Sigma; F2006) to a final concentration of 10 µg/ml; overnight at 4 degrees Celsius. Differentiated spheroids were dispersed using TripLE Express 1X (ThermoFisher Scientific; 12604021) and single cell suspension were plated on basement membrane coated dishes. Cells were cultured in maturation media S6 for 3 days in the cell culture incubator. Cells on two-well chamber slides were subsequently used for staining and imaging. Cells cultured on tissue culture treated 24-well plates were used for downstream functional assays such as glucose stimulated insulin secretion.

S1.3 | Single cell data processing and marker visualisation

Single-cell RNA-Seq data from the “stage 6” samples (GSM3141957) of human ESC (HUES8) deposited in Gene Express Omnibus were analysed. Briefly, samples with less than 200 quantified genes were filtered and genes quantified in less than three cells were excluded from downstream analysis. After filtering, expression (in counts) of each cell were normalised and scaled using Seurat R package (version 3.2.0) (PMID: 31178118) with default setting. Six different cell types were identified. Expression of different marker genes for basement membrane, fibroblast and endothelial cells were assessed using normalised data in all the six-identified cell types.
